# Knockout of TLR4 and TLR2 impair the nerve regeneration by delayed demyelination but not remyelination

**DOI:** 10.1186/1423-0127-20-62

**Published:** 2013-08-28

**Authors:** Shao-Chun Wu, Cheng-Shyuan Rau, Tsu-Hsiang Lu, Chia-Jung Wu, Yi-Chan Wu, Siou-Ling Tzeng, Yi-Chun Chen, Ching-Hua Hsieh

**Affiliations:** 1 Department of Anesthesiology, Kaohsiung Chang Gung Memorial Hospital and Chang Gung University College of Medicine, Kaohsiung, Taiwan; 2 Department of Neurosurgery, Kaohsiung Chang Gung Memorial Hospital and Chang Gung University College of Medicine, Kaohsiung, Taiwan; 3 Department of Plastic and Reconstructive Surgery, Kaohsiung Chang Gung Memorial Hospital and Chang Gung University College of Medicine, No. 123, Ta-Pei Road, Niao-Sung District, Kaohsiung City, 833, Taiwan

**Keywords:** Toll-like receptor 4 (TLR4), Toll-like receptor 2 (TLR2), Peripheral nerve regeneration, Sciatic nerve crush injury

## Abstract

**Background:**

Knockout of either toll-like receptor 4 (TLR4) or 2 (TLR2) had been reported to delay the Wallerian degeneration after peripheral nerve injury by deterring the recruitment of the macrophages and clearance of myelin debris. However, the impact on the remyelination process is poorly understood. In this study, the effect of TLR2 and TLR4 knockout on the nerve regeneration and on the remyelination process was studied in a mouse model of sciatic nerve crush injury.

**Results:**

A standard sciatic nerve crush injury by a No. 5 Jeweler forcep for consistent 30 seconds was performed in *Tlr4*^*−/−*^ (B6.B10ScN-*Tlr4*^*lps-del*^/JthJ), *Tlr2*^*−/−*^ (B6.129-Tlr2^tm1Kir^/J) and C57BL/6 mice. One centimeter of nerve segment distal to the crushed site was harvested for western blot analysis of the myelin structure protein myelin protein zero (Mpz) and the remyelination transcription factors Oct6 and Sox10 at day 0, 3, 7, 10, 14, 17, 21, 28. Nerve segment 5-mm distal to injured site from additional groups of mice at day 10 after crush injury were subjected to semi-thin section and toluidine blue stain for a quantitative histomorphometric analysis. With less remyelinated nerves and more nerve debris, the histomorphometric analysis revealed a worse nerve regeneration following the sciatic nerve crush injury in both *Tlr4*^*−/−*^ and *Tlr2*^*−/−*^ mice than the C57BL/6 mice. Although there was a delayed expression of Sox10 but not Oct6 during remyelination, with an average 4-day delay in the demyelination process, the subsequent complete formation of Mpz during remyelination was also delayed for 4 days, implying that the impaired nerve regeneration was mainly attributed to the delayed demyelination process.

**Conclusions:**

Both TLR4 and TLR2 are crucial for nerve regeneration after nerve crush injury mainly by delaying the demyelination but not the remyelination process.

## Background

Wallerian degeneration and subsequent remyelination are crucial in the nerve regeneration after peripheral nerve injury [1-3]. Upon peripheral nerve injury, the activated Schwann cells start to clean inhibitory myelin and dead neuronal debris by phagocytosis [4]. In addition, as early within 24 h after the injury [5-8], resident Schwann cells and macrophages will secret many pro-inflammatory cytokines (e.g. TNF-α, IL-1-α, IL-1β, IL-6, IL-10, and GM-CSF) and chemokines (e.g. MCP-1 and MIP-1α) to induce local inflammation and recruit more invaded macrophages for the clearance of debris. The induction process requires at least 2 days to establish an appropriate environment for the macrophage invasion [3,9]. Delay in initiation of these phenomena may not only prolong the period of disability but also impact the degree of recovery though microsurgical reconstruction [3,10]. Besides, during the process of myelination of Schwann cells in the peripheral nerve, myelin protein zero (Mpz) is abundantly expressed and regulated by some transcription factors, including Oct6, Sox10, Krox-20, c-Jun, Notch, Sox-2, Pax-3, Id2, and NF-κB [11-16]. Three transcription factors, Oct6 (SCIP/Tst1), Sox10, and Krox20 (EGR2) are considered necessary for transition from the nonmyelinating to the myelinating stage of Schwann cell development [14,15,17].

Toll-like receptors (TLRs), a family of evolutionary conserved pattern recognition receptors, act as a first line immune surveillance and priming antigen-specific adaptive immunity. TLRs can recognize infectious pathogens and trigger an innate immune response in mammals [18]. They also participate a significant role in inflammation, immune cell regulation, survival, and proliferation [19]. Certain TLRs can recognize specific endogenous molecules associated to danger signal that are released from damaged cells or tissues after injury or under stress [20,21]. TLR1, TLR2, TLR3, TLR4 and TLR7 had been discovered to be functional in Schwann cells [22,23]. Up-regulated heterodimer of TLR1/TLR2 upon stimulation and basal high level of TLR4 in both Schwann cells and sciatic nerve imply that TLR2 and TLR4 are crucial to Wallerian degeneration after peripheral nerve injury [22,23]. Boivin et al. demonstrated that *Tlr4*^*−/−*^ and *Tlr2*^*−/−*^ mice had a reduced recruitment of macrophages, persisted myelin debris in the distal nerve stump, and a significant delay of the process of Wallerian degeneration during the nerve regeneration process [9].

In this study, we are interesting in investigating the impact of the knockout of TLR2 or TLR4 gene on the nerve regeneration regarding the process of demyelination as well as remyelination. Therefore, the quantitative histomorphometric assessment of peripheral nerve architecture with detection of the time-dependent expression of Mpz, Sox10, Oct6 proteins in *Tlr2*^*−/−*^, *Tlr4*^*−/−*^ and wild type mice in a sciatic nerve crush injury were investigated in this study to answer the question.

## Methods

### Animals

Eight to twelve weeks old male mice, weighing 20-30 g were used. *Tlr2*^*−/−*^(B6.129-Tlr2^tm1Kir^/J) and *Tlr4*^*−/−*^ (B6.B10ScN-*Tlr4*^*lps-del*^/JthJ) mice were purchased from Jackson Laboratory (Bar Harbor, ME, USA). C57BL/6 mice were purchased from the National Laboratory Animal Center, Taiwan. All housing conditions were established and surgical procedures, analgesia, and assessments were performed in an AAALAC-accredited, SPF facility following national and institutional guidelines. Animal protocols were approved by the IACUC of Chang Gung Memorial Hospital.

### Peripheral nerve crush injury model

On the day of surgery (day 0), mice were anesthetized by intramuscular injection of ketamine (25 mg/kg) and xylazine (50 mg/kg). The right sciatic nerve at the mid-thigh level was exposed and then was crushed by a No. 5 Jeweler forcep for consistent 30 seconds. After release of the forcep, a 10–0 Ethilon suture (Micro suture Ethicon, Somerville, NJ) passed through the epineurium only was used to mark the injured site without constriction. For sham operated mice, the right sciatic nerve was left untouched except a mark made with epineurial suture at the corresponding site. Then all mice awaked and remained healthy in another postoperative care room.

### Western blot analysis

Four mice in each group per specific day 0, 3, 7, 10, 14, 17, 21, 28 were harvested for examination of Mpz, Oct6 and Sox10 proteins. These specimens were homogenized with tissue protein extraction reagent T-PER™ (Pierce, IL, USA) containing phosphatase and protease inhibitors. The protein samples (30 μg) were resolved on 10% SDS-polyacrylamide gels and transferred to polyvinylidenedifluoride membranes. Blots were blocked with 5% skim milk in Tween-20/ phosphate-buffered saline, and incubated with various primary antibodies as: rabbit anti–Sox10 (Millipore Biotechnology, MA, USA), anti–Oct6 (Novus Biologicals, USA), anti-Mpz (Abcam, USA) and mouse anti-β-actin (Millipore Biotechnology, MA, USA) at 4°C overnight. The blots were then incubated with horseradish peroxidase–conjugated secondary antibodies at room temperature for 60 minutes, and developed with ECL™ Western Blotting Systems (Amersham Pharmacia Biotech, Aylesbury, UK). The protein bands were quantified with FluorChem 8900 imaging system and the AlphaEaseFC software (Alpha Innotech Corp, CA, USA).

### Quantitative assessment of peripheral nerve architecture

Mice in three (*Tlr4*^*−/−*^ n = 6, *Tlr2*^*−/−*^ n = 6 and C57BL/6 n = 5) and sham-operated (C57BL/6 n = 6) groups were re-anesthetized for harvesting the studied nerve and then sacrificed at specific time point on the postoperative day 10. The axial one centimeter of nerve distal to the injured site was isolated and fixed at 4°C with 3% glutaraldehyde (Polysciences Inc., Warrington, PA, USA), washed in 0.1 M phosphate buffer (pH 7.2), post-fixed with 1% osmium tetroxide (Fisher Scientific, Pttisburgh, PA, USA), dehydrated in graded ethanol solutions, and embedded in Araldite 502 (Polysciences Inc.). Axial semithin sections, 1 μm thick, at a 5-mm distance from the injured site were stained with 1% toluidine blue for histomorphometric analysis.

We use a binary image analysis for multicomponent analysis of peripheral nerve histomorphometry [24] by an observer blinded to experimental group. Total myelinated fiber counts were measured based on six representative fields at 1000 magnification. Fiber count, fiber width, fiber area, total fiber area, fiber debris area, myelin area, axon area and axon width were calculated and analyzed.

### Statistical analysis

All the results were presented as mean ± standard error. An overall analysis of the differences between group means was calculated by one way analysis of variance (ANOVA). A post hoc Fisher’s least significant difference test was used to compare the difference between groups. In all cases, statistical significant was set at P < 0.05.

## Results

### Quantitative histomorphometric analysis

**Figure 1:**
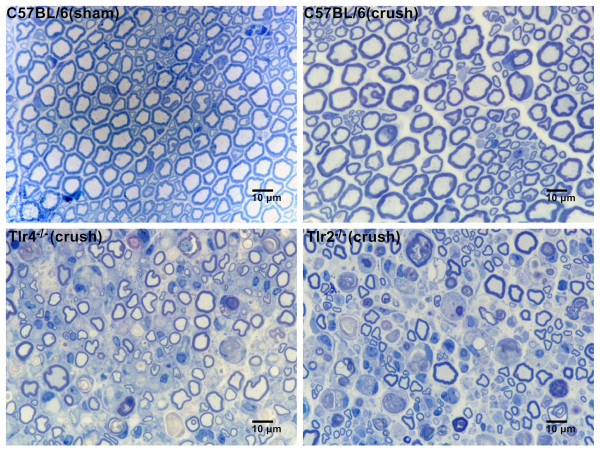
**Representative histological sections (X1000) taken distal to the nerve crush injury site on the postoperative day 10 and stained with toludine blue.** Nerve specimens were from sham-operated C57BL/6 mice as well as C57BL/6, *Tlr4*^*−/−*^, and *Tlr2*^*−/−*^ mice with nerve crush injury.

The axial section of the nerve 5 mm distal to the injured site in four mice groups (C57BL/6-sham, C57BL/6-crush, *Tlr4*^*−/−*^-crush, *Tlr2*^*−/−*^-crush) in the postoperative day 10 were illustrated in Figure 1. Analysis of the histomorphometric data was summarized in Table 1. Nerve specimens from sham-operated C57BL/6 mice display the greatest numbers of nerve fibers and intact neural tissue with the least debris. Except more fiber debris area was noted in the crushed nerve of C57BL/6 mice in comparison with those sham-operated C57BL/6 mice, there was no significant difference in all measured parameter of the histomorphometric data between these two groups of C57BL/6 mice. In *Tlr4*^*−/−*^and *Tlr2*^*−/−*^ mice, the specimens presented significant prominent debris with less percentage nerve tissue. At day 10, the fiber count, fiber width, fiber area, total fiber area, fiber debris area, myelin area, axon area and axon width in both the *Tlr4*^*−/−*^ (P < 0.05) group (*n* = 6), and the *Tlr2*^*−/−*^(P < 0.05) group (*n* = 6) were significantly smaller than in the C57BL/6 mice (*n* = 5). At the same time point, the fiber debris area of *Tlr4*^*−/−*^ and *Tlr2*^*−/−*^ mice were significantly larger than C57BL/6 mice.

**Table 1:** Quantitative histomorphometric assessment of nerve specimens 5-mm distal to crushed site of the sciatic nerve

**Group**	**n**	**Fiber count**	**Fiber width, μm**	**Fiber area, μm**^**2**^	**Total fiber area, μm**^**2**^	**Fiber debris area, μm**^**2**^	**Myelin area, μm**^**2**^	**Axon area, μm**^**2**^	**Axon width, μm**
Sham	6	112 ± 13	5.25 ± 0.35	31.5 ± 3.1	3349 ± 260	23 ± 5*	16.1 ± 1.9	12.7 ± 1.9	3.19 ± 0.36
C57BL/6	5	106 ± 10	5.34 ± 0.16	31.8 ± 2.7	3235 ± 289	51 ± 16	16.0 ± 1.4	14.3 ± 1.6	3.61 ± 0.14
Tlr4^−/−^	6	56 ± 10*	4.14 ± 0.39*	19.6 ± 3.9*	1204 ± 392*	161 ± 24*	10.5 ± 2.1*	8.6 ± 1.8*	2.68 ± 0.28*
Tlr2^−/−^	6	46 ± 6*	4.16 ± 0.26*	19.6 ± 2.4*	943 ± 191*	178 ± 38*	10.8 ± 1.4*	8.1 ± 1.0*	2.61 ± 0.16*

Data are shown as mean ± standard error (* indicated *P < 0.05* compared with C57BL/6 group).

### Expression of remyelination transcriptional factors

**Figure 2:**
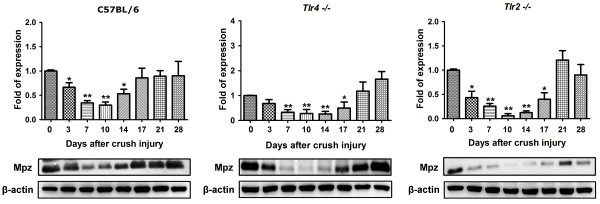
**Western blot analysis of Mpz level in day 0, 3, 7, 10, 14, 17, 21, 28 of C57BL/6, *Tlr4***^***−/−***^**and *Tlr2***^***−/−***^**mice.**(*P < 0.05;**P < 0.001).

**Figure 3:**
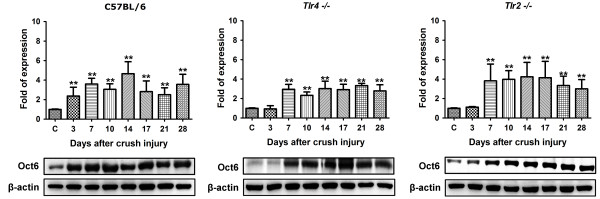
**Western blot analysis of Oct6 level in day 0, 3, 7, 10, 14, 17, 21, 28 of C57BL/6, *Tlr4***^***−/−***^**and *Tlr2***^***−/−***^**mice.**(*P < 0.05;**P < 0.001).

**Figure 4:**
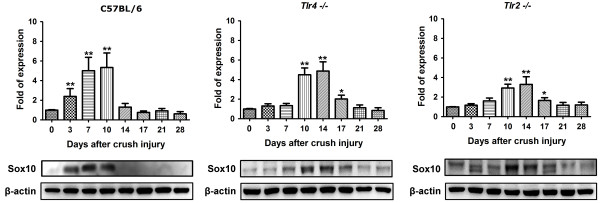
**Western blot analysis of Sox10 level in day 0, 3, 7, 10, 14, 17, 21, 28 of C57BL/6, *Tlr4***^***−/−***^**and *Tlr2***^***−/−***^**mice.**(*P < 0.05;**P < 0.001).

The Mpz of sciatic nerve of C57BL/6 mice started to decrease slightly since postoperative day 3 and returned to normal level in day 17 (Figure 2). In *Tlr4*^*−/−*^ and *Tlr2*^*−/−*^ mice, the Mpz level had a significant decrease since day 7 to day 17 with a delayed recovery in day 21. Put all together, knockout of toll-like receptor 4 or 2 signaling would contribute to the timing delay of around 4 days for the formation of the myelin structure protein. The Oct6 level (Figure 3) of C57BL/6 mice started to increase significantly since postoperative day 3. In *Tlr4*^*−/−*^ and *Tlr2*^*−/−*^ mice, the Oct6 level had a delayed increase since day 7. An average 4-day delay was also observed in Oct6 protein level of *Tlr4*^*−/−*^ and *Tlr2*^*−/−*^ mice. The Sox10 level (Figure 4) of C57BL/6 mice started to increase significantly since postoperative day 3 and returned normally in day 14. In *Tlr4*^*−/−*^ and *Tlr2*^*−/−*^ mice, the Sox10 level had the delay in significant increasing since day 10 and returned normally in day 21. A 7-day delay in activation and expression of Sox10 protein could be seen in *Tlr4*^*−/−*^ and *Tlr2*^*−/−*^ gorup when compared to C57BL/6 mice.

## Discussion

Degenerated myelin and nerve debris are both major inhibition of nerve regeneration. Without removing them successfully after Wallerian degeneration, the restoration of function won’t be initiated and achieved [22]. Both Schwann cells and macrophages can remove degenerated myelin debris without each other *in vitro*[25,26] and *in vivo*[27,28]. Clearance of degenerated myelin by resident Schwann cells and invaded macrophages is a preferable process for subsequent remyelination in the nerve regeneration. Upon axotomy, TLR1 becomes strongly induced in the peripheral nerve [22]. TLR2 signaling pathway had also been proved to involve in activation of Schwann cells [29,30]. In addition, TLR3, TLR4, and TLR7 are majorly expressed in the peripheral nerve system, with their possible role in immune surveillance [22]. In this study, we had demonstrated the nerve regeneration following the sciatic nerve crush injury in both *Tlr4*^*−/−*^ and *Tlr2*^*−/−*^ mice was worse than the C57BL/6 mice and there was significant increase of the fiber debris remained in the distal stump of the sciatic nerve. Significantly fewer macrophages were recruited and/or activated in the distal stump of sciatic nerve upon crush injury had also been reported in the TLR2-, TLR4-, and MyD88-deficient mice [9]. By using functional blocking antibodies of TLR4 and MCP-1, MCP-1 expression is impaired and induces prominent delay in macrophage recruitment and clearance of debris [6,7,29]. On the other hand, a single microinjection of TLR2 and TLR4 ligands at the site of sciatic nerve lesion had faster clearance of the degenerating myelin and recovered earlier than saline-injected control rats [9]. Although specific endogenous ligands for TLR2 or TLR4 signaling such as high mobility group box 1 protein, heat-shock proteins and extracellular matrix components is still in debate [19,31], accelerating the Wallerian degeneration might be potential to shorten the period of nerve regeneration after peripheral nerve injury [3].

Sox10 and Oct6 were discovered to be upstream positive transcriptional factors in remyelination and form the backbone of myelination promoting network [15,16]. The major target of them is Krox20 which can activate and induce expression of several meylination genes, including the major structural myelin protein (Mpz) in peripheral nerve system [32]. In addition, Sox10 is the most dominant protein in regulating Schwann cells development, especially expressed at all stages of the Schwann cell lineage [33]. Therefore, Sox10 is believed to be essential in initiation of myelination [34], throughout the myelination process [35] and in myelinating sciatic nerve *in vivo*[12]. On the other hand, the negative transcriptional factors such as c-Jun [36], Notch [16], Sox2 [37] are rapidly up-regulated following injury and suppressed as myelination starts. Moreover, some potential negative regulators, e.g. Pax-3 [38] and Id2 [39] posed paradoxically expression in myelination. The relationship and interplay of above negative regulators is very limited and obscure. Therefore, Sox10, Oct6 and MPZ were the main protein targets evaluated in this present study.

This *in vivo* study demonstrated the genetic deletion in either TLR2 or TLR4 can impact the nerve regeneration after sciatic nerve crush injury with delayed expression of myelin protein Mpz as well as the critical re-myelination transcription factors Oct6 and Sox10. In the *Tlr4*^*−/−*^ and *Tlr2*^*−/−*^ mice, there was an average 4-day delay in the expression of Mpz and Oct6 proteins and a 7-day delay in expression of Sox10 protein. With an average 4-day delay in the demyelination process, the subsequent complete formation of Mpz was delayed for 4 days. The 4-day delay expression of Oct6 may be also attributed to the 4-day delay of the demyelination process. Although there was a 7-day delay in activation and expression of Sox10 protein, there was no further impact had been found on the formation of Mpz during the remyelination.

## Conclusions

In sum, our data implied that the impaired nerve regeneration was mainly attributed to the delayed demyelination process in the *Tlr4*^*−/−*^ and *Tlr2*^*−/−*^ mice; however, the orchestrated interplay between positive and negative transcription factors to regulate proteins of myelination is much complicated and needs more studies to elucidate the mechanisms and net effect.

## Competing interests

The authors declare no potential conflict of interests.

## Authors’ contributions

SCW was responsible for the writing of the manuscript. CSR contributed to the design of animal study. THL and SLT participated in the animal surgery and acquisition of the study specimens. CJW participated in the nerve quantitative histomorphometric analysis. YCW and YCC were involved in the Western blot experiment. CHH contributed to the design of animal study and analysis and acquisition of all data. All authors read and approved the final manuscript.
